# Effects of Various Surfactants on the Dispersion of MWCNTs–OH in Aqueous Solution

**DOI:** 10.3390/nano7090262

**Published:** 2017-09-06

**Authors:** Hongzhi Cui, Xiantong Yan, Manuel Monasterio, Feng Xing

**Affiliations:** 1 Guangdong Provincial Key Laboratory of Durability for Marine Civil Engineering, College of Civil Engineering, Shenzhen University, Shenzhen 518060, China; h.z.cui@szu.edu.cn (H.C.); yanxiantong@email.szu.edu.cn (X.Y.); perokeseto@protonmail.com (M.M.); 2 Shenzhen Advanced Civil Engineering Technology, Association Research Center, Shenzhen Institute of Information Technology, Shenzhen 518172, China; 3 State Key Lab of Advanced Welding Production Technology, Harbin Institute of Technology Shenzhen Graduate School, Shenzhen 518055, China

**Keywords:** carbon nanotube, surfactant, dispersibility, stability, ultrasonic, UV–Vis, surface modification

## Abstract

Dispersion of carbon nanotubes (CNTs) is a challenge for their application in the resulting matrixes. The present study conducted a comparison investigation of the effect of four surfactants: Alkylphenol polyoxyethylene ether (APEO), Silane modified polycarboxylate (Silane-PCE), I-Cationic polycarboxylate (I-C-PCE), and II-Cationic polycarboxylate (II-C-PCE) on the dispersion of hydroxyl functionalized multi-walled carbon nanotubes (MWCNTs–OH). Among the four surfactants, APEO and II-C-PCE provide the best and the worst dispersion effect of CNTs in water, respectively. Dispersion effect of MWCNTs–OH has been characterized by optical microscope (OM), field emission-scanning electron microscope (FE-SEM), and Ultraviolet–visible spectroscopy (UV–Vis).The OM images are well consistent with the UV–Vis results. Based on the chemical molecular structures of the four surfactants, the mechanism of MWCNTs–OH dispersion in water was investigated. For each kind of surfactant, an optimum surfactant/MWCNTs–OH ratio has been determined. This ratio showed a significant influence on the dispersion of MWCNTs–OH. Surfactant concentration higher or lower than this value can weaken the dispersion quality of MWCNTs–OH.

## 1. Introduction

Carbon Nanotubes (CNTs) are regarded as a one-dimensional carbon nanomaterial. According to the layer number, they are classified as either single-walled carbon nanotubes (SWCNTs) or multi-walled carbon nanotubes (MWCNTs). CNTs can be defined as rolled up from a single planar sheet of graphene, in the case of SWCNTs, and multi planar sheets in MWCNTs. Since the CNTs were discovered occasionally by Iijima in 1991 [1], great interests have been attracted by its superb mechanical [2], electric [3], thermal [4], and optical [5] properties, and various applications in bio-sensors [6], composites [7], field emission devices [8], energy or gas storage [9], and probe tips [10] etc.

CNTs are extremely hydrophobic, and due to the delocalization of π–electrons, CNTs exhibit very significant conductivity, besides, the adsorption between CNTs and different chemical moieties are also enhanced [11]. In addition, CNTs have a large aspect ratio, which makes them easy to entangle and bundle with strong van der Waals interaction [12,13]. Particularly, the energies of tube-tube contact by van der Waals are as high as 500 eV/μm [14]. Thus, for such high interaction energy, it is a challenging task to disperse CNTs uniformly.

Mechanical methods and chemical approaches are most widely used in dispersing CNT agglomerations. Mechanical methods, like ultra-sonication, high shear mixing, and ball milling are usually used to shorten CNTs lengths by inputting high energy or direct mechanical contact [15,16]. Among these three methods, ultra-sonication is the most typical technique for dispersing CNTs in water, which exfoliates the CNTs by exposing them to cavitation. High shear stresses generated by cavitation, can de-bundle CNT clusters easily. However, the ultra-sonication induced dispersion was found, in previous works, to be reversible in the short term [17]. Thus, the chemical approaches include covalent and non-covalent methods. Covalent methods are characterized by functionalization with a variety of chemical moieties such as HNO_3_/H_2_SO_4_, ozone, etc. to improve the solubility of CNTs in solvents. Nevertheless, an aggressive chemical functionalization creates defects on the surface of CNTs and, consequently, an alteration in the CNTs properties [18]. A non-covalent method is characterized by the π–π stacking interaction, electronic attraction or van der Waals interaction between the chemical moieties and the nanotube surface. Generally, a non-covalent approach is thought to be superior than covalent methods as it has little influence on the π–electron cloud of CNTs, while preserving the excellent properties of attaching a variety of chemical groups on CNTs surfaces [19].

Through non-covalent adsorption on CNTs surfaces, surfactants (SAA) enable the CNTs to preserve the dispersing state for a long period in aqueous solutions, therefore altering the environmental behavior and utilization of CNTs. To date, the CNT dispersibility of various surfactants and corresponding acting mechanisms have been investigated by a variety of researches {I.E. Octyl-phenol-ethoxylate (Triton X-100) [20], Sodiumdodecylsulfate (SDS) [21], Dodecyl trimethylammonium bromide (DTAB) [22], Sodium dodecylbenzenesulfonate (NaDDBS) [23], etc.}. The MWCNTs contain –COOH functional group and commercial polyacrylate based surfactant containing particular types of polycarboxylate chains was used. In 2015, Zou, et al. [24] investigated the absorbance of different CNT concentration under varied ultrasonication energy (UE) by adding a commercially available polycarboxylate-based cement superplasticizer. Results of the study indicated that absorbance (ABS) gradually increased with respect to UE and almost proportional to the concentration, and an ABS plateau was reached at an ultrasonication energy of 250 J/mL. According to these studies, the formation of a layer of a surfactant coat on CNT surface contributes to counterbalance van der Waals attractions between CNTs by electrostatic and/or steric repulsions. In this sense, the dispersion and agglomeration of CNTs are regulated by a thermodynamic equilibrium that was created by the balance between repulsive and attractive forces. The adsorption between surfactants and CNTs can be affected by a number of factors. At present, influencing factors like alkyl chain length [25], charge concentration [26], or the interactions between coated surfactants and the functional groups on CNTs have been investigated.

This study has carried out a comparative analysis of MWCNTs–OH dispersion by four different surfactants: Alkylphenol polyoxyethylene ether (APEO), Silane modified polycarboxylate (Silane-PCE), I-Cationic polycarboxylate (I-C-PCE) and II-Cationic polycarboxylate (II-C-PCE). All selected surfactants in this research are superplasticizers for cement. Therefore, the study results can be utilized for further studies on CNTs modified cement based composites. Due to the hydroxyl (–OH) of MWCNTs–OH can form chemical bonds with cement hydration production, therefore, MWCNTs–OH rather than pristine MWCNTs was used in this study. The dispersing abilities of the surfactants have been studied experimentally and theoretically, assuming of their chemical structure. The experimental results provide insight into some of the parameters for further optimization of MWCNTs–OH dispersion using surfactants. Visual observation and UV–Vis spectroscopy have been applied to analyze the dispersibility of each surfactant. Through this study, the importance of employing a certain ratio of surfactant and MWCNTs–OH can be established, in order to obtain an optimum dispersion.

## 2. Materials and Methods

### 2.1. Materials

**Figure 1:**
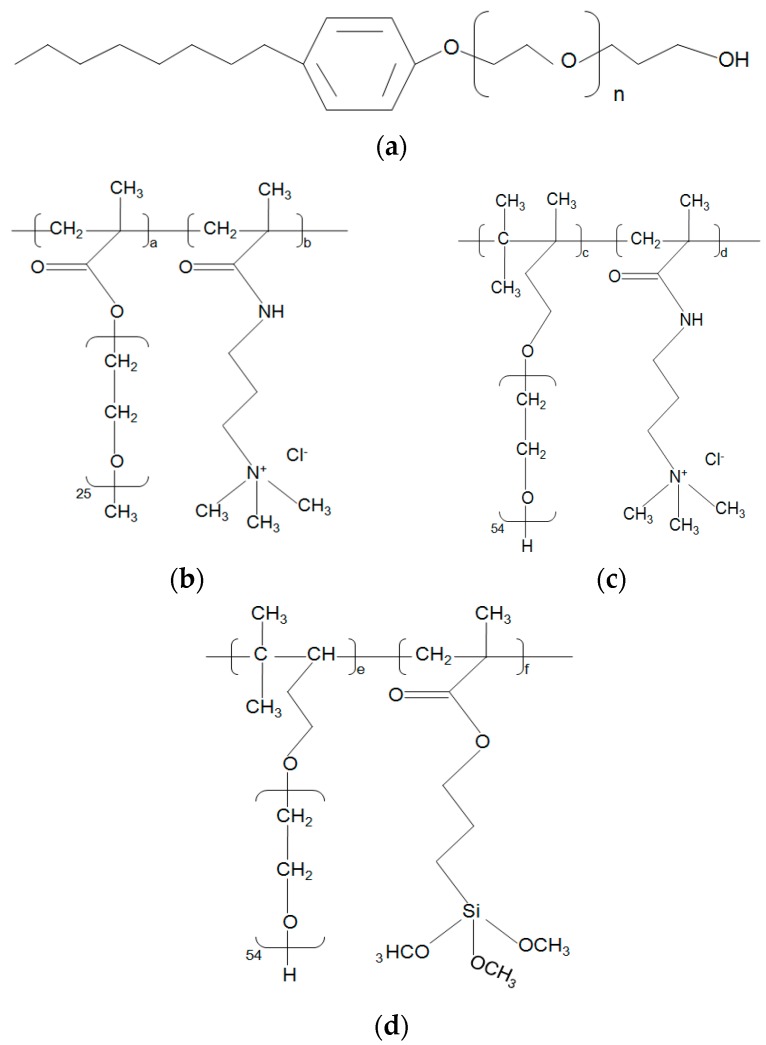
Chemical molecular structure of four surfactants. (**a**) Alkylphenol polyoxyethylene ether (APEO); (**b**) I-Cationic polycarboxylate (I-C-PCE) and (**c**) II-Cationic polycarboxylate (II-C-PCE); (**d**) Silane modified polycarboxylate (Silane-PCE).

MWCNTs with –OH functional group were purchased from Chengdu Organic Chemicals Co., Ltd., Chengdu, China, Chinese Academy of Sciences. It was produced by a chemical vapor deposition (CVD) device. An acid treatment was introduced to purify the raw MWCNTS–OH, as according to the manufacturer. Acid oxidation has been washed away before the CNTs were released. The main physical properties of MWCNTs–OH are shown in Table 1. APEO, Silane-PCE, I-C-PCE, and II-C-PCE were used as surfactants to disperse MWCNTs–OH in de-ionized water, and their chemical structures are shown in Figure 1; the four surfactants are amphiphilic composed by identical hydrophobic and hydrophilic head groups. APEO was brought from Shenzhen Benno Industrial Co., Ltd., Shenzhen, China. While Silane-PCE, I-C-PCE and II-C-PCE were synthesized in our own laboratory.

### 2.2. Preparation of MWCNTs–OH Dispersion with Various Surfactant

In order to compare the dispersing capacity of the four surfactants and establish the optimal CNT to surfactant ratio for each surfactant, dispersions of surfactants were prepared at concentrations spanning from 0.1 to 14 g/L, according to the category of surfactant and keeping the concentration of MWCNTs–OH (1 g/L) constant. The MWCNTs–OH and surfactant were mixed previously with 100 mL of de-ionized water into a flask and stirred for 15 min. Then, the dissolution was performed with an ultrasonication treatment with a 650 W ultrasonic cell crusher (Scientz-IID, Ningbo, China), set to amplitude of 30% and in 4 s cycles, for the sake of protecting the mixture from being over-heated. The whole ultrasonication time was 30 min. After the ultrasonication had finished, the dispersion of MWCNTs–OH were diluted 100 times and measured by visual observation and UV–Vis spectrometry. In the UV–Vis measurement, each sample was measured three times and the average absorbance values, at 600 nm of the UV–Vis spectrum, were used for following comparison. 

### 2.3. Characterization

#### 2.3.1. Characterization of Raw MWCNTs–OH

The identification of the phase, present in the raw materials was made by X-ray diffraction (XRD), using a Brucker D8 ADVANCE equipment (Bruker, Madison, WI, USA) in a 2θ interval between 10° and 70°, with a steps of 0.02°. Morphology of the as-received carbon nanotubes was examined using Hitachi Su-70 field emission-scanning electron microscope (FE-SEM, Tokyo, Japan) at an accelerating voltage of 20.0 kV, the MWCNTs–OH were sprayed with gold before observation. Fourier Transform Infrared Spectrometer (FT-IR) experiments were conducted to identify the functional groups on the surface of MWCNTs–OH and the four surfactants. The FT-IR model used was a Nicolet Nexus670 Fourier Transform Infrared Spectrometer (Nicolet, Madison, WI, USA), in transmission mode with KBr pellets.

#### 2.3.2. Characterization of MWCNTs–OH Dispersion

Suspensions containing a well-known ratio of MWCNTs–OH and surfactant samples were prepared by ultrasioncation of MWCNTs–OH in an aqueous solution with the different surfactants selected. After the ultrasonication process, a pipette tip was carefully used to suck up 0.5 mL of the suspension and dipped it in a glass vial, which contained 100 mL of deionized water. The morphologies of the suspensions were directly examined by naked-eye observation.

After the macroscopic observation, microscopic views of the dispersing state of MWCNTs–OH with different surfactants were obtained by conducting analyses on Hitachi Su-70 field emission-scanning electron microscope (FE-SEM) (Hitachi High-Technologies Corporation, Tokyo, Japan). Samples were prepared by drop coating MWCNTs–OH solutions on a clean side glass and drying at 50 °C in an oven, followed by the coating of a thin layer of gold before observation.

Later the microscopic observation, the dispersion of MWCNTs–OH produced by the different surfactants, was measured by UV–Vis spectrometry. A PerkinElmer Lambda 750 spectrophotometer (PerkinElmer, Waltham, MA, USA) with a wavelength range from 190 to 1100 nm was used. During the experiment, pure solutions of the four surfactants were measured as baseline correction to the substrate, their absorbance values from that of MWCNTs–OH dispersions.

## 3. Results and Discussion

### 3.1. Characterization of Raw MWCNTs–OH

**Figure 2:**
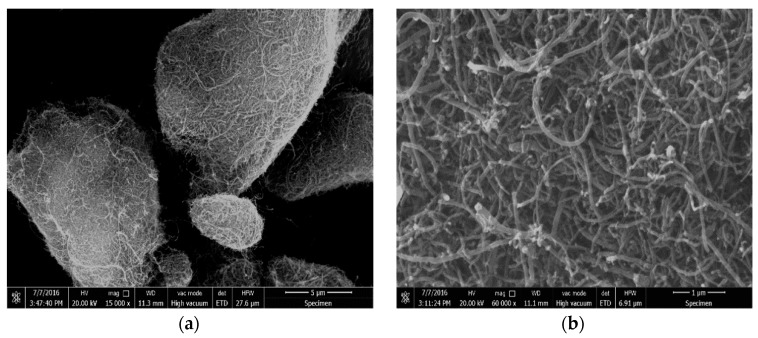
SEM images of hydroxyl functionalized multi-walled carbon nanotubes (MWCNTs–OH) under different magnification. (**a**) 15,000×; (**b**) 60,000×.

SEM was employed to study the morphology of the MWCNTs–OH before ultrasonication treatment. The SEM photograph (Figure 2) showed the micro-morphology of MWCNTs–OH under different magnifications. It can be easily found that MWCNTs–OH agglomerated with each other into ball-like bundles, as it is shown in Figure 2a. In a higher magnification (60,000×), the morphology of individual MWCNTs–OH is so clear that defective sites and nano-particles are readily found on the surface of MWCNTs–OH, as it can be found in Figure 2b.

**Figure 3:**
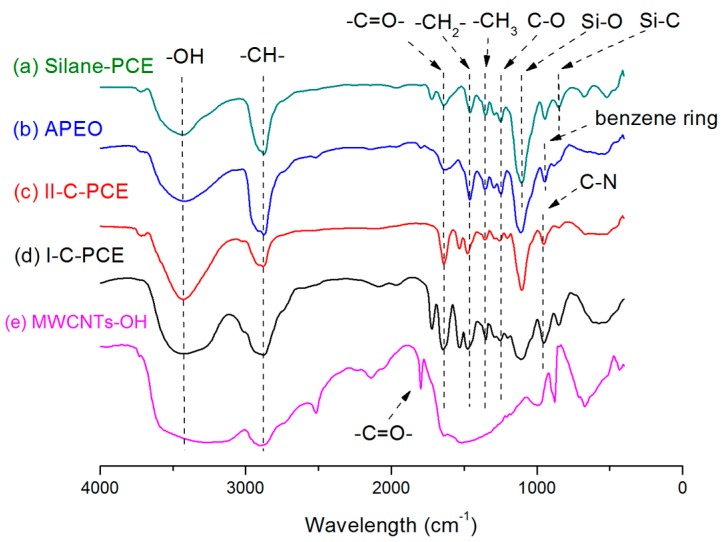
Fourier Transform Infrared Spectrometer (FTIR) spectra of MWCNTs–OH and four surfactants.

The feature of the functionalized groups in MWCNTs–OH and four surfactants were both confirmed by FT-IR spectroscopy. The FT-IR spectra of raw MWCNTs–OH and surfactants are demonstrated in Figure 3. Curve (e) is the FT-IR spectrum of pristine MWCNTs–OH, while curve (a), (b), (c), and (d) represent the Silane-PCE, APEO, II-C-PCE, and I-C-PCE, respectively. As it can be seen from Figure 3, a wide band near 3400 cm^−1^ was found in the five curves, which is commonly attributed to the –OH stretching [27]. The strong peak at about 2850 cm^−1^ is largely assigned to the symmetrical stretching vibration mode of –CH– [28,29]. The asymmetrical bending vibration of –CH3 was found at 1350 cm^−1^ [30]. Peak appearing at 1465 cm^−1^ is strongly related to the –CH_2_– (alkane bending) [27]. The sharp band at 1797 cm^−1^ in the FT-IR spectrum of MWCNTs–OH, and the peak around 1640 cm^−1^ in the FT-IR spectra of four surfactants correspond to –C=O– stretching vibration [31,32,33]. Another small peak around 1210 cm^−1^, represents the C–O stretching vibration [34]. The sharp band at 945 cm^−1^ in curve (b) is due to the nature C–C stretching of benzene ring. At curve (c) and curve (d), the strong peak at 950 cm^−1^ is associated with C–N vibration [35]. In the case of curve (a), the bands at 1099 cm^−1^ and 840 cm^−1^ are assigned to the Si–O and Si–C stretching vibration [36], respectively.

The FT-IR spectra suggested that the MWCNTs used in this experiment were hydroxyl functionalized and there were carboxyl functionalized groups on their surfaces. It is because when the fresh MWCNTs were acid treated to remove the catalyst particles, the carbon atoms on defective sites, or the tips of the tube, were oxidized into carboxyl (–COOH) [37]. The functional groups traced in the FT-IR spectrum are consistent with the chemical structure of the four surfactants. 

**Figure 4:**
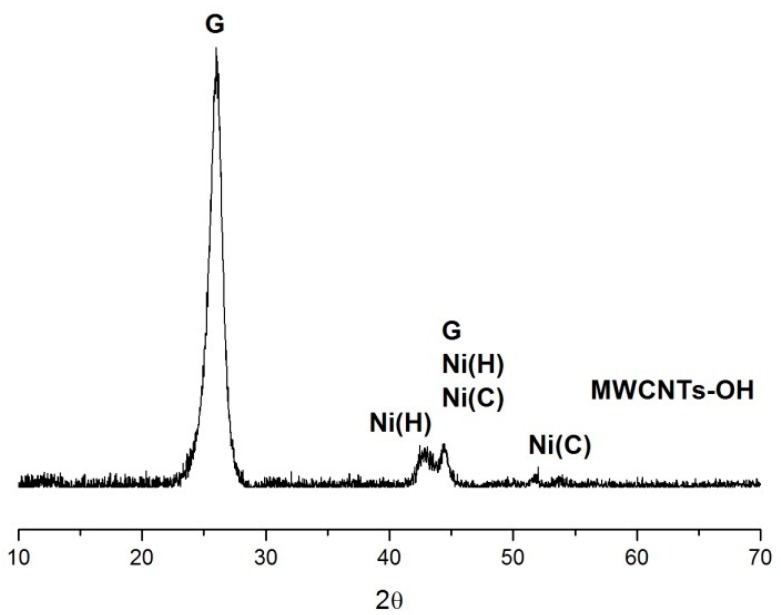
X-ray diffraction (XRD) pattern of MWCNTs–OH (G: Graphite, Ni(C): Cubic Nickel, Ni(H): Hexagonal Nickel).

XRD measurement was carried out to identify the crystalline phases present in the raw materials. As it can be seen from the XRD result (Figure 4), the strong peak of graphite is attributed to that of highly oriented pyrolytic graphite. The weak intensity of the metallic nickel and nickel oxide indicated that the content of impurities from the production is relatively low. Obviously, the nano-particle detected in the SEM analysis is assigned to the nano-nickel particles, which is used as a catalyst in the CNTs production procedures.

### 3.2. Characterization of MWCNTs–OH Dispersions

The ideal dispersion will support the most MWCNTs–OH and exhibit acceptable stability with the lowest amount of surfactant used.

#### 3.2.1. Visual Observation

**Figure 5:**
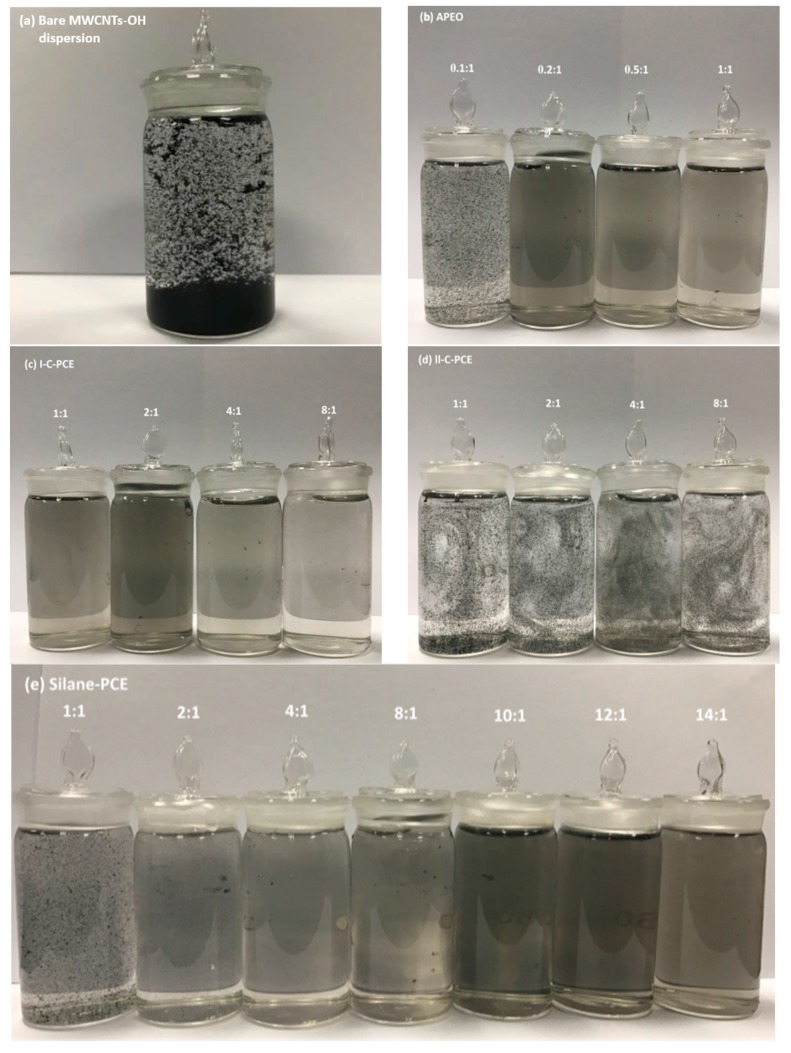
Photographs of MWCNTs–OH suspensions with different surfactants and ratios of surfactant/MWCNTs–OH: (**a**) bare MWCNTs–OH suspension; (**b**) APEO; (**c**) I-C-PCE; (**d**) II-C-PCE; (**e**) Silane-PCE.

Images taken for visual observation are shown in Figure 5a–e. Although single MWCNTs–OH cannot be resolved by optical microscopy, nevertheless, it enables the directly characterization for the dispersing state of MWCNTs–OH suspension on a micrometer scale. Figure 5a shows the image of MWCNTs–OH suspensions without surfactant, where it can be found MWCNTs–OH aggregates and dense clusters in the bottom of the vial after ultasonication treatment. It seems obvious that it is impossible the direct dispersion of MWCNTs–OH in water with non-active surfaces. However, the addition of a surfactant makes it different, as it can be seen in Figure 5b–e. The CNT suspensions stabilized with surfactants, showed a remarkable change in their agglomeration state; seeing from the images of some surfactant-aid-suspensions, only a few carbon agglomerations can be found, suggesting that most of the MWCNTs–OH have been dispersed. By varying the categories of the surfactant and the ratio of surfactant/MWCNTs–OH, homogeneous and single-phase suspensions were obtained, but some of the suspensions were still heterogeneous. 

#### 3.2.2. SEM Analyses

**Figure 6:**
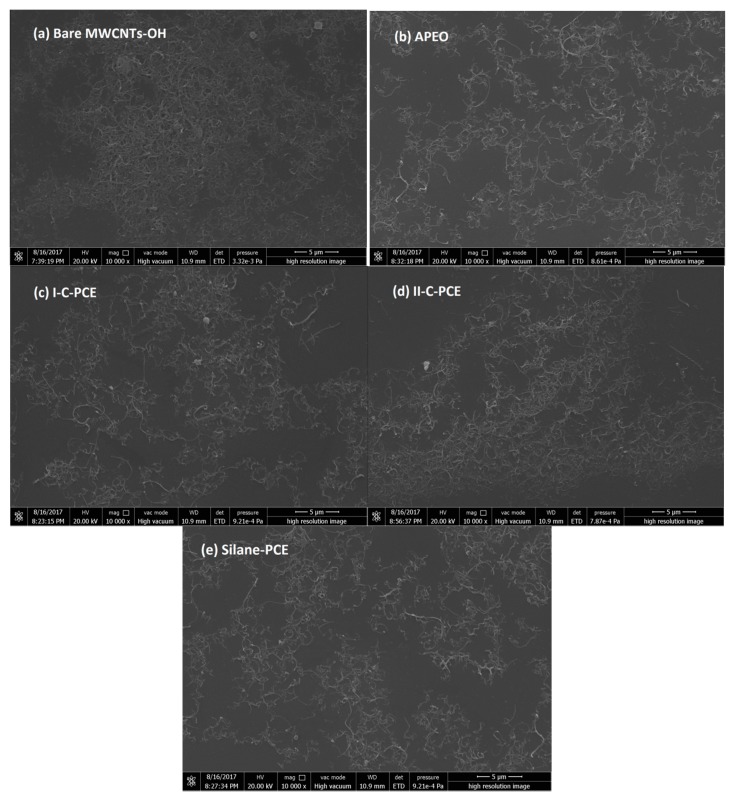
SEM images of MWCNTS–OH with different surfactant. (**a**) Bare MWCNTs–OH; (**b**) APEO; (**c**) I-C-PCE; (**d**) II-C-PCE; and (**e**) Silane-PCE.

Figure 6 shows typical FE-SEM images of dispersed MWCNTs–OH with and without surfactants. In comparison with the bare MWCNTs–OH in Figure 6a, surfactant aids MWCNTs–OH to exhibit remarkable improvement in terms of dispersing state. While MWCNTs–OH clusters can be easily found in Figure 6a, MWCNTs–OH with surfactant are well dispersed and uniformly distributed in Figure 6b–e. As it can be seen, the density of MWCNTs–OH in Figure 6d is higher than that of Figure 6b,c,e, which indicating that APEO, I-C-PCE, Silane-PCE show stronger dispersibility than II-C-PCE. However, the similarities of dispersion among APEO, I-C-PCE, and Silane-PCE of the MWCNTs–OH, suggest that further investigations should be conducted to distinguish the difference in dispersibility of APEO, I-C-PCE, and Silane-PCE.

### 3.3. Comparison of Various MWCNTs–OH Dispersions Using UV–Vis Spectroscopy

In the UV–Vis spectra, individual MWCNTs–OH can be identified by the π–π transition of the –C=C–, which shows a strong peak at 270 nm [5,38]. CNT aggregates, however, are not activated under the same irradiation, because carriers are tunneling between the nanotubes [39]. Due to the water dispersion over the surface modified of MWCNTs–OH well obeys the Lambert-Beer law, the absorbance at 600 nm [40] is proportional to the dispersed CNTs. Actually, ambient conditions of CNTs have little influence on the absorbance at this wavelength. Therefore, the UV–Vis results indicate to us the amount of dispersed MWCNTs–OH in an aqueous solution [18,19,20].

**Figure 7:**
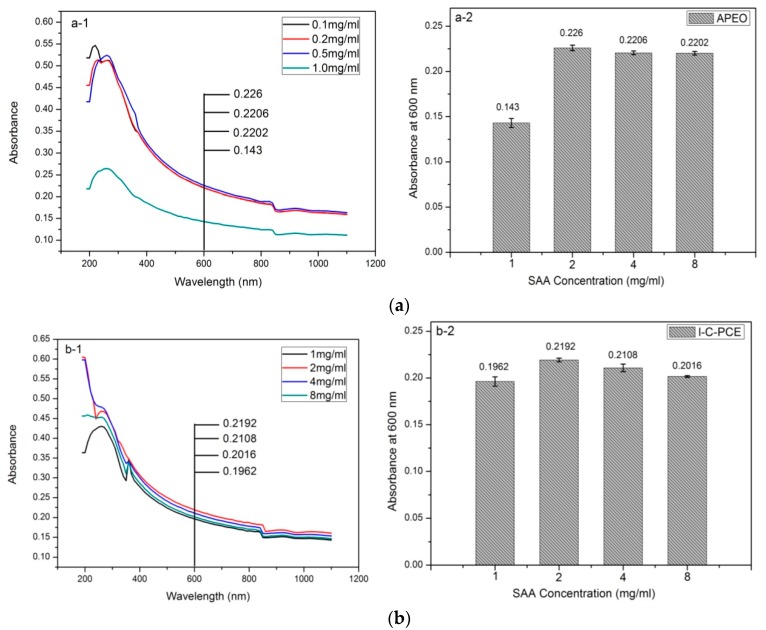

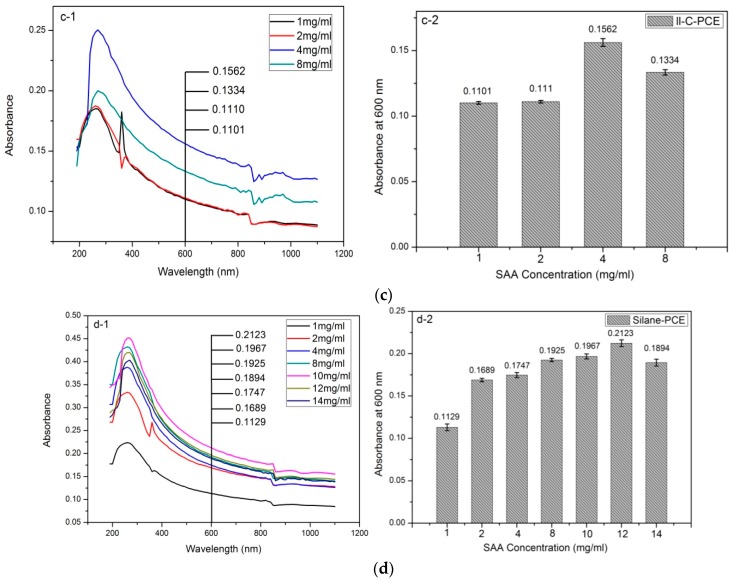
UV–Vis spectra of MWCNTs–OH dispersions with (**a**) APEO; (**b**) I-C-PCE; (**c**) II-C-PCE; and, (**d**) Silane-PCE, and absorbance at 600 nm vs. different SAA concentrations.“1”in the figures illustrates the UV–Vis spectra of MWCNTs–OH suspensions manufactured by varying categories and concentration of surfactant solutions; “2” in the figures demonstrates the Lambert-Beer absorbance value at 600 nm depend on the concentration of the surfactants.

Figure 7 illustrates the UV–Vis spectra of MWCNTs–OH suspensions manufactured by varying categories and concentration of surfactant solutions. The figure at the right side of the UV–Vis spectra demonstrates the Lambert-Beer absorbance value at 600 nm depend on the concentration of the surfactants. It is noticeable that the absorbance values of MWCNTs–OH suspension at 600 nm vs. surfactant concentration for all the surfactants follows a Gaussian trend, which exhibits an increasing trend when the surfactant/MWCNTs–OH ratio is increased, until the maximum absorbance is achieved. Any surfactant/MWCNTs–OH ratio below or higher than this value will deteriorate the homogeneity of MWCNTs–OH suspensions. For the subsequent increase in the surfactant/MWCNTs–OH ratio, the absorbance of CNT suspensions at 600 nm decreased as the amount of individual CNT in the suspension declined due to the reduction of electrostatic repulsion forces or the steric hindrance effect between CNTs. The reason for this might be when at a low surfactant/MWCNTs–OH ratio, insufficient surfactant is unable to form an efficient coating which induces the electrostatic repulsion or steric hindrance effect to counter-balance the van der Waals attractions [30]. However, when excessive surfactants are introduced into the suspensions, the formation of micelles [41] will directly increase the osmotic pressure of the aqueous system and, subsequently, creates an effective attraction, because the micelles cannot fit the gaps between the adjacent CNTs. As a consequence, individual CNT re-agglomerates in the aqueous solution. This attraction is referred to, both in classical colloidal suspensions [42] and in multiwall nanotube dispersions [43], as depletion attraction [44]. Eventually, an optimal surfactant/MWCNTs–OH ratio value is obtained for supporting the maximum amount of individual CNT with just a sufficient amount of surfactant. This maximum value of absorbance for the APEO, I-C-PCE, II-C-PCE, and Silane-PCE were 0.226, 0.2192, 0.1562, and 0.2123 when the surfactant/MWCNTs–OH ratio was 1:5, 2:1, 4:1, and 12:1, respectively. Thus, the same amount of APEO can disperse the largest amount of MWCNTs–OH, as compared to the others three surfactants. Although I-C-PCE and Silane-PCE showed little differences, in terms of absorbance, I-C-PCE proved to be better as it requires fewer amounts of surfactant to disperse the same amount of MWCNTs–OH. Therefore, the dispersing ability of the four surfactants follows the order: APEO > I-C-PCE > Silane-PCE > II-C-PCE, according to the experiment realized.

### 3.4. Relationship between Dispersibility and Chemical Molecular Structure of Surfactant

The above observed differencesin dispersibility among the four surfactants can also be illustrated by their chemical structure, as shown in Figure 1. When the surfactant is employed to disperse the CNTs in water, the surfactant molecules tend to orient themselves on the surface of CNTs, with hydrophobic tail groups facing toward CNTs while hydrophilic tail groups face toward the water phase under the driving force of hydrophobic interaction or electrostatic force, which results in a decrease in the interfacial tension of CNTs/water. Therefore, the dispersibility of the surfactant is closely related to how strongly it absorbs on the surface of CNTs, and sufficient height of energy barriers that produce by these adsorptions, which prevent individual CNT from re-agglomeration [45]. The results of some researches indicated that the benzene ring structure in surfactant molecules proved to enhance the adsorption between surfactant molecular and graphitic surface via π–π stacking type interaction [11,46,47]. It is generally recognized that graphitic unit cells match well with methylene units of hydrocarbon chains [48], leading to hydrophobic tail groups orienting themselves flat on the surface of CNTs.Hence, the primary adsorption mechanism can be attributed to the hydrophobic interaction and π–π interactions between the surfactants and CNTs. In this circumstance, the adsorption efficiency and the dispersibilities of surfactants are significantly influenced by the tail length of the surfactant, for the reason that longer tails bring about a higher spatial volume and a greater steric hindrance, which provides a stronger repulsive forces between CNTs [45].

It is known, as it can be checked in Figure 1, that Silane-PCE and II-C-PCE have the longest hydrocarbon tail length in the side chain of the molecular unit, while APEO has the shortest hydrocarbon tail among the four surfactants. Therefore, theoretically, APEO should show minimum dispersibility and Silane-PCE or II-C-PCE should exhibit the maximum dispersibility, contrary to the empirical obtained results, as exposed in Figure 6. Such a contradiction is probably attributed to the existence of a benzene ring structure in the chemical molecular structure of APEO. As it is above mentioned, the benzene ring structure enables surfactant molecules to absorb more strongly the graphitic surface through the π–π stacking interaction [45]. Except for the “benzene ring” factor, it is easier for surfactants with shorter branches to penetrate into the gaps in the CNT clusters when ultrasonication is applied [49]. Furthermore, according to Bai’s research [50], surfactants with a shorter hydrophilic chain length exhibit greater adsorption capacities, and because of the same reason, I-C-PCE exhibits a greater dispersing ability than II-C-PCE, due to this hydrophilic chain length difference in their chemical molecular structure (see Figure 1). In total, APEO provides the best capacity among the four surfactants with respect to dispersed MWCNTs–OH in water.

When we have compared I-C-PCE with II-C-PCE, in terms of dispersing ability, I-C-PCE shows a greater dispersing potential, due to the mentioned “hydrophilic chain length factor”, which is the same in the APEO surfactant. As a result, for a surfactant with a shorter hydrophilic chain length, it is less possible to form a micelle that is caused by an interaction between the side chains of the neighboring surfactant molecular, which would cause a depletion reduction effect, and consequently, lead to the re-agglomeration of MWCNTs–OH in an aqueous solution [45]. Therefore, a large amount of MWCNTs–OH clusters can be found in II-C-PCE surfactant results, as shown in Figure 5d. However, when it comes to I-C-PCE and Silane-PCE, the former one exhibits a greater dispersing ability not only because of the “hydrophilic chain length factor”, but also because of the significant enhancement in the adsorption between MWCNTs–OH and I-C-PCE, as induced by the electrostatic attractions between the negative charged MWCNTs–OH [51] and cationic I-C-PCE molecular. Besides, owning to the extra electrostatic repulsions that are provided by I-C-PCE’s cationic head group at the side chain, the ability of the system to counterbalance the van der Waals attractions between MWCNTs–OH improved to a large extent [52]. As it can be observed in Figure 6(d-1,d-2), I-C-PCE exhibits a maximum dispersing ability with an optimal surfactant/CNTs ratio of 2:1, while Silane-PCE needs twelve times more mass of MWCNTs–OH to reach its desirable dispersing ability, indicating the fact that Silane-PCE possesses a lower adsorption efficiency and a weaker ability to stabilize the dispersion than I-C-PCE. As a result, I-C-PCE shows a better dispersing ability than Silane-PCE and II-C-PCE.

The chemical molecular structure of Silane-PCE and II-C-PCE is similar, with the same hydrophilic chain length in their branches. The most characteristic difference between the two surfactants is the head group in the side chains. Silane-PCE has trialkoxysilane groups Si–(OCH_3_)_3_ at the end of the side chains while II-C-PCE has a N–(CH_3_)_3_ group in the same position. According to Fan’s research [53], trialkoxysilane groups have a strong impact on the absorbed amount of Silane-PCE on MWCNTs–OH. After it is dissolved in water, the siloxane groups in the side chain of silane-PCE could form covalent bonds with MWCNTs–OH, via a dehydration condensation reaction, which significantly improves both the absorbed amount of Silane-PCE on MWCNTs–OH and the adsorption force between the two components. Generally, a covalent bond between MWCNTs–OH and silane-PCE is stronger than the adsorption that is driven by electrostatic attraction or van der Waals attractions between MWCNTs–OH and II-C-PCE. Therefore, Silane-PCE displays a greater dispersing ability than II-C-PCE. The aforementioned findings prompt us to conclude that, when the “hydrophilic chain length factor” and “siloxane groups” compete between themselves, the former contributes more to the dispersion of MWCNTs–OH; however, the latter shows a vast advantage when it is compared with “electrostatic attraction factor” in terms of adsorption.

## 4. Conclusions

In this study, comparative experiments have been conducted to study the difference in the dispersing capacities of four surfactants, namely APEO, Silane-PCE, I-C-PCE, and II-C-PCE. The main task of this research was to study the factors that are responsible for the dispersing capacities of the four surfactants, as well as the difference between them. With this key idea, visual observations and UV–Vis studies of MWCNTs–OH dispersion in above surfactant solutions were executed. The experimental results have indicated that the dispersing ability shows the following trend: APEO > I-C-PCE > Silane-PCE > II-C-PCE. This tendency was further checked according to the characteristics of chemical molecular structures of the four surfactants. The experimental investigation was consistent with the results of some researches, which suggest that APEO benefits come from its benzene ring, when it intents to disperse MWCNTs–OH in aqueous solution. Besides, another finding enables us to make the conclusion that the “hydrophilic chain length factor” contributes more to MWCNTs–OH dispersion in comparison to the “siloxane group factor”. Thus, the chemical molecular structure of a surfactant has a great impact on its dispersing ability. The main purpose of this research is the obtaining of the optimum surfactant/MWCNTs–OH ratio. This ratio has great significance, due to the quality of MWCNTs–OH suspensions deteriorating when this ratio was lower or higher than the optimum value. Thereby, in order to avoid any excess that would decrease the quality of MWCNTs–OH dispersion, the surfactant concentration should be controlled to be just sufficient to coat the MWCNTs–OH surface. It was found, in our study, that nanotube dispersions obtained the best quality when the surfactant/MWCNTs–OH ratio for APEO, I-C-PCE, Silane-PCE, and II-C-PCE was 0.2:1, 2:1, 12:1, and 4:1, respectively. In conclusion, when choosing a surfactant to disperse MWCNTs–OH in aqueous solution, factors like chemical molecular structure and the ratio of surfactant/MWCNTs–OH ratio should be taken into consideration.

## Figures and Tables

**Table 1:** Physical properties of MWCNTs–OH.

Type	OD	–OH Content	Length	Purity	Ash	SSA	EC
MWCNTs–OH	>50 nm	0.76%	20 μm	>90 wt %	<6 wt %	>40 m^2^/g	>102 s/cm

Note: OD means Outer Diameter; SSA means Specific Surface Aare; EC means Electrical Conductivity.
